# DNAJC3-AS1 Is Associated with Proliferation, Metastasis, and Poor Prognosis of Breast Cancer

**DOI:** 10.1155/2021/3443474

**Published:** 2021-11-03

**Authors:** Yi Zhang, Jing-jing Li, Bo Luo, Xiao-fei Guo, Jian-xin Liu, Shun-shi Yang

**Affiliations:** ^1^Department of Ultrasound, The Central Hospital of Wuhan, Tongji Medical College, Huazhong University of Science and Technology, Wuhan, Hubei, China; ^2^Department of Pathology, The Central Hospital of Wuhan, Tongji Medical College, Huazhong University of Science and Technology, Wuhan, Hubei, China

## Abstract

**Objective:**

Long noncoding RNA DNAJC3-AS1 (DNAJC3-AS1) was a newly identified tumor-related lncRNA. The aim of the present study was to explore the prognostic value and diagnostic of DNAJC3-AS1 (DNAJC3-AS1) expression in breast cancer (BC) patients. *Patients and Methods*. The expression of DNAJC3-AS1 was detected in 170 BC tissues and matched normal breast samples by qRT-PCR. The diagnostic value of DNAJC3-AS1 was examined by receiver-operating characteristic (ROC) assays. The correlation of DNAJC3-AS1 with clinicopathological features and prognosis was also statistically analyzed. CCK-8 assays, colony formation assays, and Transwell assays were applied to examine the potential function of DNAJC3-AS1 on tumor progression. Western blot was used to examine the expression of EMT-related proteins.

**Results:**

The expression of DNAJC3-AS1 in BC specimens was higher than that in the adjacent nontumor tissues (*p* < 0.01). Diagnostic assays revealed that DNAJC3-AS1 has considerable diagnostic accuracy, with an area under the ROC curve (AUC) of 0.7457 (*p* < 0.001). High DNAJC3-AS1 expression was positively associated with lymph node metastasis (*p* = 0.010) and clinical stage (*p* = 0.023). A survival study revealed that patients with high DNAJC3-AS1 expression had shorter overall survival (*p* = 0.0067) and disease-free survival (*p* < 0.0001) than those with low DNAJC3-AS1 expression. More importantly, multivariate assays indicated that DNAJC3-AS1 was an independent prognostic factor in BC patients. Functional assays confirmed that silence of DNAJC3-AS1 distinctly suppressed the proliferation, metastasis, and EMT progress of BC cells.

**Conclusions:**

DNAJC3-AS1 may be a prognostic and diagnostic biomarker for BC patients.

## 1. Introduction

Breast cancer (BC) is the most common type of cancer and the main type of cancer that leads to the death of females around the world [1]. It was estimated that 1.65 million cases would be diagnosed with BC and the death toll would reach 535,800 in 2018 [2]. In China, BC, with an increasing incidence on a yearly basis, seriously threatens the health and even the life of females [3]. In recent decades, people have made a lot of efforts to find out the molecular mechanism regulating BC genesis. Despite the big contribution of chemotherapeutic agents to the obvious reduction of the mortality of BC patients, those in an advanced state still hold a poor survival because of the aggressive clinical behavior [4, 5]. Therefore, the identification of novel molecules that are involved in the pathogenesis and progression of BC is essential for the improvement of BC diagnostic and therapy.

More than 70% of human genomes are under active transcription, while protein-coding genes only occupy 12% of human genomes [6]. Noncoding RNAs (ncRNAs) constitute most transcripts, which include long ncRNAs (lncRNAs). lncRNAs belong to a class of ncRNAs with the length over 200 nucleotides [7, 8]. lncRNAs regulate different levels of gene expression to participate in various biological processes including differentiation, angiogenesis, immune responses, and apoptosis [9]. More and more studies have reported the role of lncRNAs in the initiation and development of tumors as oncogenes and/or tumor suppressors [10]. Besides, the abnormal expression profiles of lncRNAs in clinical BC specimens can affect the grade of malignancy as well as the histological differentiation, exhibiting significant clinical implications for diagnosing cancer subclassification [11, 12]. Based on these findings, lncRNAs can be potentially used to diagnose and treat tumors as a key target [13, 14]. Thus, the identification of novel lncRNAs involved in the progression of BC was urgent and necessary.

lncRNA DNAJC3-AS1, located on 13q32.1, was a novel tumor-related lncRNA which was firstly demonstrated to be highly expressed in osteosarcoma and promote the proliferation and metastasis by its sense-cognate gene DNAJC3 [15]. Studies have confirmed that DNAJC3 could help cells fight against the endoplasmic reticulum stress so to suppress cellular apoptosis, thus influencing cellular biological response, such as cellular damages or programmed cell death [16]. Then, a distinct upregulation of DNAJC3-AS1 was also observed in colon cancer [17]. However, the expression pattern and function of DNAJC3-AS1 in other tumors remained largely unclear. In this study, we firstly provided evidences that DNAJC3-AS1 expression was distinctly upregulated in BC patients and predicted a poor prognosis. In addition, we confirmed that knockdown of DNAJC3-AS1 suppressed the proliferation, migration, invasion, and EMT progress of BC cells. Our findings suggested DNAJC3-AS1 as a prognostic and therapeutic target for BC patients.

## 2. Patients and Methods

### 2.1. Patients and Tissue Samples

Tumor tissues with paired adjacent normal tissues were obtained from 170 BC patients at the Central Hospital of Wuhan, Tongji Medical College, Huazhong University of Science and Technology, from July 2013 to August 2016. All those patients had not underwent radiotherapy, chemotherapy, or endocrine treatment prior to surgery. All samples, after quick-freezing, were stored at -80°C for qRT-PCR. The clinical examination, together with the histopathological analysis on tissue specimens, helped to make a diagnosis. Patients' characteristics are described in Table 1. All BC patients were followed for 60 months. The study has obtained the approval from the Ethics Committee of the Central Hospital of Wuhan, Tongji Medical College, Huazhong University of Science and Technology, and obtained patients' informed consent before beginning. The study was carried out following the Helsinki Declaration.

### 2.2. Cell Lines and Transfection

Human BC cell lines ZR-75-1, MCF-7, BT-20, MDAMB-231, and SKBR3 and an immortalized breast epithelial cell line MCF-10A were purchased from American Type Culture Collection (ATCC, Manassas, VA, USA). All cells were cultured in RPMI-1640, supplemented with 10% FBS at 37°C in a humidified atmosphere of 5% CO_2_. Small interfering RNA of DNAJC3-AS1 and the negative control siRNA were obtained from Sigma-Aldrich (USA). According to the manufacturer's protocol, cells were transfected with Lipofectamine 2000 (Invitrogen).

### 2.3. Quantitative Real-Time PCR

RNAiso Plus (Takara, Suzhou, Jiangsu, China) was used to extract total RNA from cells or tissues, and HiFiScript cDNA Kit (Invitrogen, Haidian, Beijing, China) was used to reverse-transcribe the extracted total RNA into cDNA. ND-1200 NanoDrop spectrophotometer (NanoDrop, Shenzhen, Guangzhou, China) determined the concentration and purity of RNA at the absorbance at 280 nm. The miScript Reverse Transcription together with miScript SYBR Green PCR Kit assisted in performing the real-time quantitative RT-PCR, following the protocol of manufacturers (Foster City, CA, USA). GAPDH was used as an endogenous control. The fold changes were calculated with the relative quantification (2^−*ΔΔ*Ct^) method. All reactions were run in triplicate. Invitrogen (Haidian, Beijing, China) provided the primers for PCR. The primer sequences were as follows: DNAJC3-AS1, forward (5′-AGCGATTGTGGAAGACCTCC-3′) and reverse (5′-ATTTCCCCTGGTACACGGCT-3′); GAPDH, forward (5′-GGTGA AGGTCGGAGTCAACGC-3′) and reverse (5′-CAAAGTTGTCATGGAG-3′).

### 2.4. Proliferation Cell Counting Kit-8 (CCK-8) Assays

CCK-8 assays were conducted by the use of a CCK-8 assay kit (Abbexa, Aimei Technology, Hubei, China). The transfected cells were seeded into culture plates, followed by the treatments of 10 *μ*L of CCK-8 reagent. We measured absorbance at 450 nm.

### 2.5. Colony Formation Assay

Cells were transfected with si-DNAJC3-AS1 or si-NC, at a density of 400 cells/6 cm dish. The above cells were trypsinized and counted. After the surviving cells developed colonies, 3.7% methanol was used to fix colonies, followed by 0.1% crystal violet. Colonies containing at least 50 cells were scored.

### 2.6. Transwell Migration and Invasion Assays

24-well transwell chambers with an 8 *μ*m pore polycarbonate membrane insert were used to study the effects of DNAJC3-AS1 knockdown on metastasis ability of BC cells. 4 × 10^5^ cells were loaded onto the upper compartment. To the lower compartment, 500 *μ*L medium was added. A chamber coated with Matrigel (Corning, Pudong, Shanghai, China) was applied for invasion assays. Giemsa (Solarbio, Hangzhou, Zhejiang, China) was applied for the stain of the invasive and migrative cells. A light microscope was used to count the cells.

### 2.7. Western Blot

Firstly, 20 *μ*L proteins were separated by 10% SDS-PAGE (Beyotime, Hangzhou. Zhejiang, China). Subsequently, 0.5 *μ*m PVDF membranes (Millipore, Hangzhou. Zhejiang, China) were applied for the transfection of the above proteins. After blocking with skim milk, the membranes were incubated overnight at 4°C with anti-E-cadherin, anti-N-cadherin, anti-Vimentin, and anti-GAPDH antibodies. Then membranes were incubated with a horseradish peroxidase-conjugated secondary antibody for 1 hour. Enhanced chemiluminescence was applied to detect the signal. All antibodies are purchased from Abcam (Pudong, Shanghai, China).

### 2.8. Statistical Analysis

Analyses were performed using GraphPad Prism (Prism 6) and R 3.6.1 software. The Student *t*-test or the chi-square test helped examine whether the difference was statistically significant between groups. Kaplan-Meier survival curves evaluated the survival, and the survival difference between groups was examined by the log-rank test. Multivariate analyses on the prognostic values were performed with the help of a Cox proportional hazard regression analysis. A *p* value less than 0.05 was considered with statistical significance.

## 3. Results

### 3.1. DNAJC3-AS1 Is Upregulated in Human BC Tissues

**Figure 1:**
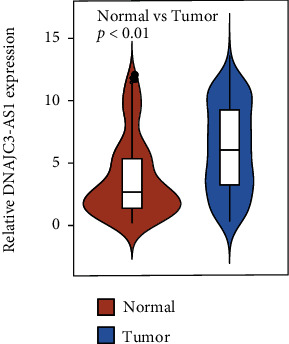
Expression of DNAJC3-AS1 is increased in BC tissues compared with noncancerous tissues through qRT-PCR (*p* < 0.01). Student's *t*-test was used for the comparison.

For confirming DNAJC3-AS1 expression pattern in BC tissues, we collected 170 pairs of BC specimens and matched normal tissues from 170 BC patients in our hospital and further performed RT-PCR. As presented in Figure 1, our group observed that relative DNAJC3-AS1 levels were distinctly higher in BC tissues than those in normal breast specimens (*p* < 0.01).

### 3.2. The Possible Diagnostic Significance of DNAJC3-AS1 Expression for BC Patients

**Figure 2:**
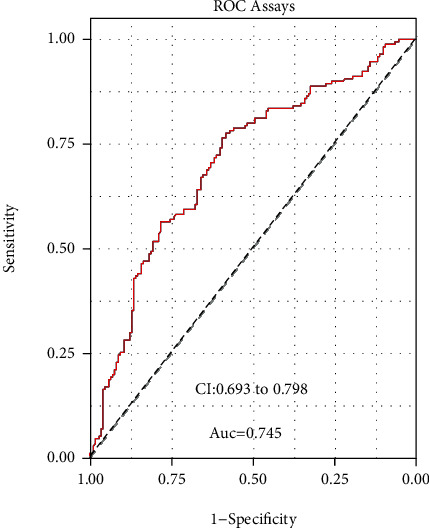
ROC curve for diagnostic value of DNAJC3-AS1 in BC patients.

Given the distinct overexpression of DNAJC3-AS1 in BC patients, we wondered whether DNAJC3-AS1 could have a diagnostic value. According to the ROC assays, our group observed that high DNAJC3-AS1 expression had an AUC value of 0.7457 (95% CI: 0.6934 to 0.7980) for BC (Figure 2). The ideal cut-off value was 5.55. Our data suggested that DNAJC3-AS1 may be an indicator for the diagnosis of BC patients.

### 3.3. Association of DNAJC3-AS1 Expression with Clinicopathological Features of BC Patients

Previous studies have reported that several lncRNAs can assist in regulating the clinical progression of various tumors. Thus, the possible clinical significance exhibited by DNAJC3-AS1 expression in BC patients was explored. The mean expression levels of DNAJC3-AS1 (8.36) were taken into account for dividing patients with BC into a group with a high expression level and a group with a low expression level. Table 1 listed such relations. High DNAJC3-AS1 expression was observed to lead to positive lymph node metastasis (*p* = 0.010) as well as advanced clinical stage (*p* = 0.023). Nevertheless, DNAJC3-AS1 expression exhibited no obvious relation to other clinicopathological characteristics (all *p* > 0.05).

### 3.4. Prognostic Value of DNAJC3-AS1 Expressions for BC Patients

**Figure 3:**
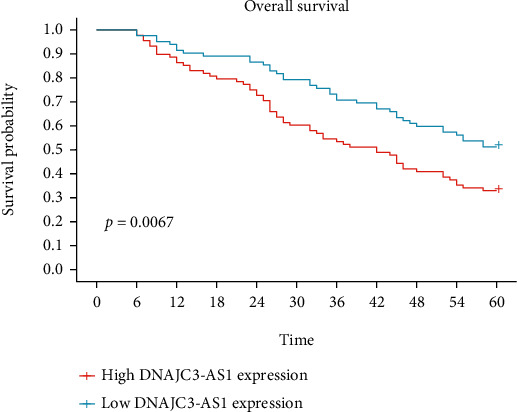
Kaplan-Meier assays for the overall survival in relation to DNAJC3-AS1 expression level in 170 patients with BC.

**Figure 4:**
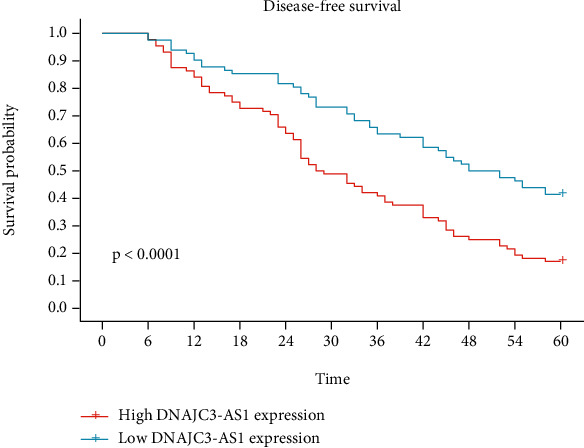
Kaplan-Meier assays for the disease-free survival in relation to DNAJC3-AS1 expression level in 170 patients with BC.

In recent years, more and more studies have reported the potential of lncRNAs used as novel prognostic biomarkers. Then, associations between the DNAJC3-AS1 expression and five-year survivals were evaluated by Kaplan-Meier survival assays. Importantly, we observed that patients whose DNAJC3-AS1 expression was high exhibited a shorter OS (*p* = 0.0067; Figure 3) and disease-free survival (DFS, *p* < 0.0001; Figure 4) as compared with the DNAJC3-AS1-low group. Moreover, multivariate assays suggested that high DNAJC3-AS1 expression was independently associated with OS (HR = 2.956, 95% CI: 1.285-4.892, *p* = 0.003) and DFS (HR = 3.228, 95% CI: 1.328-5.218, *p* = 0.001). Our finding revealed that increased DNAJC3-AS1 expression may be a biomarker of unfavorable outcomes for BC patients (Table 2).

### 3.5. Silence of DNAJC3-AS1 Promoted BC Progression

**Figure 5:**
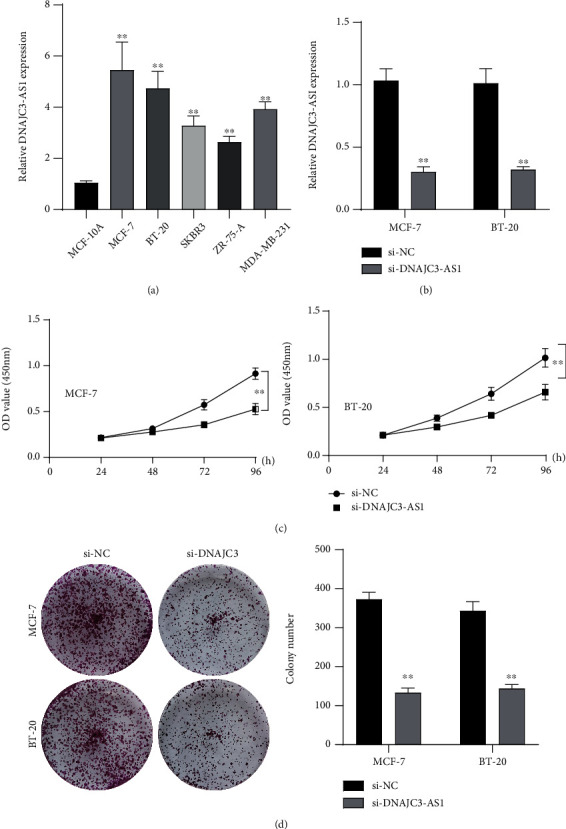
Knockdown of DNAJC3-AS1 suppressed the proliferation of BC cells. (a) The relative DNAJC3-AS1 expression was detected in five BC cells compared to MCF-10A. (b) DNAJC3-AS1 was silenced by si-DNAJC3-AS1 in MCF-7 and BT-20 cells. (c, d) CCK-8 and colony formation assays of BC cells with silencing of DNAJC3-AS1. Differences between two groups were analyzed by *t*-test. ^∗∗^*p* < 0.01.

**Figure 6:**
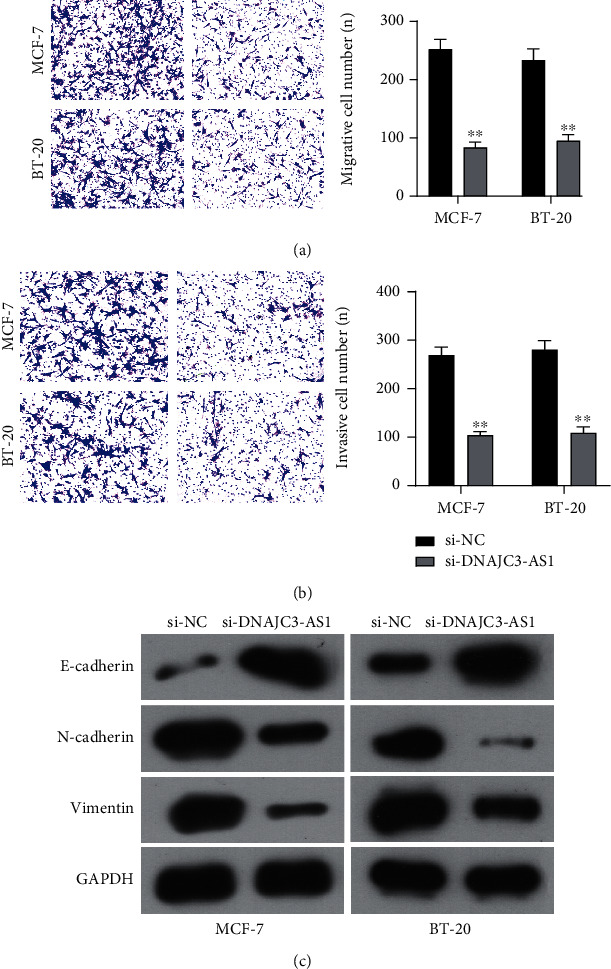
DNAJC3-AS1 knockdown inhibited the metastasis of BC cells. (a) After transfection with indicated siRNAs, migrative abilities of MCF-7 and BT-20 cells were determined using Transwell assays. (b) DNAJC3-AS1 knockdown inhibited the invasive abilities of MCF-7 and BT-20 cells. (c) The expressions of Vimentin, N-cadherin, and E-cadherin in transfected MCF-7 and BT-20 cells were detected by Western blot. Differences between 2 groups were analyzed by *t*-test. ^∗∗^*p* < 0.01.

To study the functions of DNAJC3-AS1 in BC, we firstly performed RT-PCR to examine the expression of DNAJC3-AS1 in several BC cells. As shown in Figure 5(a), we found that DNAJC3-AS1 expression was distinctly increased in five BC cell lines (MCF-7, BT-20, ZR-75-1, MDAMB-231, and SKBR3) compared with MCF-10A cells. siRNAs against different DNAJC3-AS1 were performed, which effectively knocked down DNAJC3-AS1 expression level in MCF-7 and BT-20 cells (Figure 5(b)). Further CCK-8 assays and colony formation assay confirmed that knockdown of DNAJC3-AS1 distinctly suppressed the proliferation of MCF-7 and BT-20 cells (Figures 5(c) and 5(d)). Furthermore, we explored whether DNAJC3-AS1 was involved in cell metastasis in MCF-7 and BT-20 cells. Transwell migration assays suggested that DNAJC3-AS1 silence distinctly decreased migration abilities in MCF-7 and BT-20 cells (Figure 6(a)). Applying invasion assays, our group reported that the number of invaded cells was distinctly reduced in the DNAJC3-AS1 knockdown cells (Figure 6(b)). Moreover, we conducted Western blot assays to detect the expressions of EMT-associated proteins after DNAJC3-AS1 silence.  Figure 6(c) indicated that knockdown of DNAJC3-AS1 distinctly promoted the expressions of E-cadherin while inhibiting the expressions of N-cadherin and Vimentin in both MCF-7 and BT-20 cells.

## 4. Discussion

BC, one of the most common malignant tumors, is becoming the most threatened killer of women health in the world [5]. Different patients have obviously different survival time. As the gene expression and cytology present heterogeneity, coordinating high-efficient therapeutic strategies suitable for all patients faces some difficulty [18, 19]. The application of sensitive diagnostic and prognostic biomarkers for BC patients was very important for the clinical outcome of BC. Up to date, several clinical factors have been widely used in clinical detection of BC patients, and novel potential biomarkers have been also developed, such as dysregulated genes involved in tumor progression, methylation, and various ncRNAs [20, 21]. The study paid attention to identifying novel BC-related lncRNAs.

Recently, more and more researchers have paid attention to the ability of lncRNAs to remarkably regulate the transcription process of genes. In BC, several lncRNAs have been functionally identified. For instance, lncRNA SNHG3, an overexpressed lncRNA in BC, promote BC cell proliferation and invasion via modulating miR-384/HDGF axis [22]. LINC01121, a newly identified tumor-related factor, was reported to be highly expressed in both BC specimens and the cell lines and exhibit a tumor-promotive role by promoting BC cell proliferation and invasion via regulating miRNA-150-5p/HMGA2 [23]. These findings suggested several functional lncRNAs as potential tumor suppressors or oncogenes in BC, highlighting their clinical application used as novel diagnostic and prognostic biomarkers for BC. In recent years, a new lncRNA, DNAJC3-AS1, attracted our attention. In osteosarcoma, DNAJC3-AS1 expression was found to be distinctly increased. Functional assays revealed that DNAJC3-AS1 knockdown suppressed the proliferation and invasion via sense-cognate gene DNAJC3 [15]. Then, Han and his group reported that DNAJC3-AS1 expression presented a distinct upregulation in colon cancer, and its knockdown suppressed the migration and EMT progress of the colon cancer cell via miRNA-214-3p/LIVIN axis [17]. These findings revealed that DNAJC3-AS1 is a potential oncogene in tumors. However, whether DNAJC3-AS1 was dysregulated in BC and its clinical significance in tumor patients, including BC, has not been investigated.

In this study, we firstly found an increase in the DNAJC3-AS1 expression in BC patients, which was consistent with its overexpression in colon cancer and osteosarcoma. Then, ROC assays revealed that high DNAJC3-AS1 expression showed a great diagnostic value (AUC = 0.745; sensitivity, 82.39%-100%; specificity, 73.24%-93%) in BC patients, suggesting DNAJC3-AS1 as a potential factor. Moreover, clinical research revealed that increased DNAJC3-AS1 expressions may result in advanced clinical stage, indicating that it may contribute to the clinical progress of BC. In a survival study, we observed that patients whose DNAJC3-AS1 expression is higher exhibited a relatively shorter OS and DFS, which was confirmed by analyzing the survival of 170 patients with five-year follow-up. More importantly, multivariate assays confirmed that DNAJC3-AS1 expression could be used to independently predict the BC prognosis regarding the 5-year OS and DFS. Finally, we confirmed that knockdown of DNAJC3-AS1 distinctly suppressed the proliferation, migration, invasion, and EMT progress of BC cells. Thus, our findings firstly provided clinical evidence that DNAJC3-AS1 expression was distinctly upregulated in BC and may be used as a novel biomarker and therapeutic target for BC patients. However, large clinical trials are needed to further demonstrate the above results due to the small sample size in our cohort.

## 5. Conclusions

We firstly showed that the expression profile of DNAJC3-AS1, an oncogenic lncRNA, might serve as a prognostic and diagnostic marker in patients with BC. Further studies are needed to examine the mechanisms underlying the oncogenic function of DNAJC3-AS1 for this malignancy.

## Figures and Tables

**Table 1:** The correlations between DNAJC3-AS1 expression and clinicopathological factors of BC patients.

Characteristics	*N*	DNAJC3-AS1 expression		*p*
		High	Low	
Age (years)				0.775
≤50	81	41	40	
>50	89	47	42	
Tumor size (cm)				0.259
≤2	94	45	49	
>2	76	43	33	
Differentiation grade				0.950
G1 + 2	102	53	49	
G3	68	35	33	
Histological type				0.493
Ductal	97	48	49	
Lobular	73	40	33	
ER status				0.609
Negative	94	47	47	
Positive	76	41	35	
PR status				0.150
Negative	94	44	50	
Positive	76	44	32	
HER-2 status				0.397
Negative	98	48	50	
Positive	72	40	32	
Lymph node metastasis				0.010
Absent	112	50	62	
Present	58	38	20	
Clinical stage				0.023
I + II	101	45	56	
III	69	43	26	

**Table 2:** Multivariate survival analysis of overall survival and disease-free survival in 170 BC patients.

Variables	Overall survival			Disease-free survival		
	RR	95% CI	*p*	RR	95% CI	*p*
Age	0.895	0.472-1.665	0.217	0.938	0.623-1.894	0.185
Tumor size	1.217	0.774-2.184	0.137	1.443	0.582-2.331	0.118
Differentiation grade	1.341	0.675-2.211	0.176	0.986	0.776-1.978	0.328
Histological type	1.544	0.784-2.019	0.132	1.492	0.998-1.987	0.247
ER status	0.897	0.472-1.896	0.337	1.127	0.632-2.018	0.132
PR status	1.137	0.663-2.123	0.216	1.328	0.853-2.289	0.137
HER-2 status	0.986	0.527-1.897	0.238	1.132	0.773-2.218	0.198
Lymph node metastasis	3.254	1.327-6.237	0.001	3.582	1.498-7.382	0.001
Clinical stage	3.018	1.227-4.786	0.008	3.237	1.426-5.334	0.002
DNAJC3-AS1 expression	2.956	1.285-4.892	0.003	3.228	1.328-5.218	0.001

## Data Availability

The data used to support the findings of the present study are available from the corresponding author upon reasonable request.
